# Supplementary material for: Hybrid Models and Biological Model Reduction with PyDSTool
Source: PLoS Comput Biol. 2012 Aug 9;8(8):e1002628. doi: 10.1371/journal.pcbi.1002628 (PMC3415397; doi:10.1371/journal.pcbi.1002628)
Supplement: Text S4 — Complete source code for the PyDSTool package (version 0.88.120504). Includes API documentation and help files linking to web pages. This file is identical to the current public release on Sourceforge.net. (ZIP) [file pcbi.1002628.s004.zip › PyDSTool/html/toc-PyDSTool.PyCont.Continuation-module.html]

xml version="1.0" encoding="ascii"?


Continuation


# Module Continuation

---

## Classes

Continuation  
 EquilibriumCurve  
 FixedPointCurve  
 FoldCurve  
 HopfCurveOne  
 HopfCurveTwo  
 LimitCycleCurve  
 UserDefinedCurve  

## Functions

random

## Variables

FLOQ\_TOL  

Inf

NaN

PI  

\_classes

\_constants

all\_args\_list  
 all\_curve\_types  
 all\_point\_types  
 argmax  
 auto\_point\_types  
 bif\_curve\_colors  
 bif\_point\_colors  

c\_

clock  
 conjugate  
 cont\_args\_list  
 cont\_bif\_points  

divide

equilibrium\_args\_list  
 equilibrium\_bif\_points  
 evec1\_standard  
 fixedpoint\_args\_list  
 fixedpoint\_bif\_points  
 fold\_args\_list  
 fold\_bif\_points  
 hopf\_args\_list  
 hopf\_bif\_points  
 identity  

isfinite

isnan

limitcycle\_args\_list  
 limitcycle\_bif\_points  

log10

mod

multiply  
 ndarray  
 negate  
 other\_special\_points  
 pointsToPointset  

r\_

sign

solution\_measures  
 solution\_measures\_list  
 sqrt  
 stab\_line\_styles  

subtract

sum  
 userdefined\_args\_list  

---

[hide private]
